# Sialylation as an Important Regulator of Antibody Function

**DOI:** 10.3389/fimmu.2022.818736

**Published:** 2022-04-07

**Authors:** Ravi Vattepu, Sunny Lyn Sneed, Robert M. Anthony

**Affiliations:** Center for Immunology and Inflammatory Diseases, Division of Rheumatology, Allergy and Immunology, Department of Medicine, Massachusetts General Hospital, Harvard Medical School, Boston, MA, United States

**Keywords:** antibody, glycosylation, sialylation, *in vitro* glycoengineering, *in vivo* glyco engineering, sialic acid, Immunoglobulins

## Abstract

Antibodies play a critical role in linking the adaptive immune response to the innate immune system. In humans, antibodies are categorized into five classes, IgG, IgM, IgA, IgE, and IgD, based on constant region sequence, structure, and tropism. In serum, IgG is the most abundant antibody, comprising 75% of antibodies in circulation, followed by IgA at 15%, IgM at 10%, and IgD and IgE are the least abundant. All human antibody classes are post-translationally modified by sugars. The resulting glycans take on many divergent structures and can be attached in an N-linked or O-linked manner, and are distinct by antibody class, and by position on each antibody. Many of these glycan structures on antibodies are capped by sialic acid. It is well established that the composition of the N-linked glycans on IgG exert a profound influence on its effector functions. However, recent studies have described the influence of glycans, particularly sialic acid for other antibody classes. Here, we discuss the role of glycosylation, with a focus on terminal sialylation, in the biology and function across all antibody classes. Sialylation has been shown to influence not only IgG, but IgE, IgM, and IgA biology, making it an important and unappreciated regulator of antibody function.

## Introduction

Antibodies are critical mediators of to host defense and homeostasis. Further, the therapeutic potential of antibodies has been fundamental in advancing basic immunology and biotechnology. Antibodies have two unique functions that are responsible for their biological function. They can recognize specific structures with high affinity and specificity, and simultaneously engage with cells of the innate immune system. Antibodies can be passively transferred across individuals or species, which gives them therapeutic utility. Indeed, polyclonal IgG from tens of thousands of healthy individuals, intravenous immunoglobulin (IVIG), is administered as IgG replacement therapy to immunocompromised individuals, and as an anti-inflammatory agent to some patients suffering from inflammatory and autoimmune diseases (1–3). Hybridoma technologies enable generation of monoclonal antibody preparations. The molecular biology revolution following the invention of PCR enabled cloning and recombinant production of monoclonal antibodies. Currently, there are 100 monoclonal antibodies (mAbs) approved by the FDA and monoclonal antibodies are the fastest growing class of therapeutics (4).

**Figure 1:**
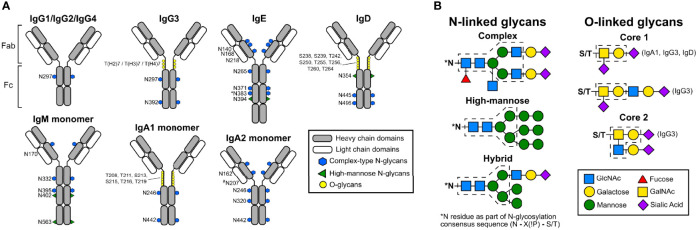
Schematic representations of human antibody structures and attached glycans. **(A)** Human immunoglobulin structures with corresponding glycosylation sites (human numbering) are shown. Each antibody isotype is comprised identical heavy chains (gray) and light chains (white), forming the antigen-binding fragment **(Fab)** and fragment crystallizable region (Fc). While monomeric structures are shown, IgM typically multimerizes into a pentamers or hexamers, and IgA1 and IgA2 can form dimers. Blue hexagons denote sites occupied by complex-type N-glycans. Green triangles indicate sites occupied by oligomannose N-glycans. Yellow circles mark sites occupied by O-linked glycans. The N383 site on human IgE is unoccupied, and N207 on human IgA2 is present only in specific allotypes. **(B)** Structures of N- and O-linked glycans found on human antibodies are represented. N-linked glycans are attached to asparagine residues containing the consensus sequence N-X-S/T. N-linked glycans can form complex, high-mannose, or hybrid types. Blue squares; GlcNAc. Yellow circles; galactose. Green circles; mannose. Red triangles; fucose. Yellow squares; GalNAc. Purple diamonds; sialic acid. Dashed lines indicate core structures, and sugar residues outside the core are variable additions.

The monomeric structures of antibodies are composed of two identical heavy chains and two identical light chains connected by disulfide bonds (5, 6). In humans, antibodies are categorized into five classes: IgG, IgM, IgA, IgE, and IgD, based on constant region structure, properties, and oligomerization (**Figure 1A**) (7). In serum, IgG is the most abundant antibody comprising 75% of all antibodies in circulation, followed by IgA at 15%, IgM at 10%, and IgD and IgE are the least abundant (6, 8). These antibodies are structurally different and elicit different effector functions through interactions between their fragment crystallizable (Fc) portion and different Fc receptors (6).

Therapeutic antibodies currently used in the clinic belong only to the IgG class because of their functional characteristics such as long half-life, strong antigen binding, and effector functions such as antibody-dependent cellular cytotoxicity (ADCC) and complement-dependent cytotoxicity (CDC). There is interest in developing therapeutic IgM, IgE, and IgA antibodies to explore novel targets and harness the effector functions of different immune cells. In contrast to IgG antibodies, IgM antibodies do not bind to Fc gamma receptors (FcγRs), but do bind the Fc mu receptor (FcμR) and can trigger potent CDC activity by binding to C1q (9). Currently, there are some IgM antibodies in clinical development (9). Further, IgE-based therapeutic antibodies are being evaluated in different studies (10). Advantages of using IgE include higher affinity interactions with the Fc epsilon receptor I (FcϵRI), longer tissue residence time, and ability to infiltrate the tumor microenvironment (10). Similarly, the development of IgA therapeutics poses advantages such as the ability to recruit neutrophils to kill tumors cells, targeting IgA interactions with the Fc alpha receptor FcαRI, and mucosal applications (11). However, there are numerous challenges in the expression, purification, and characterization of IgE, IgM, and IgA, including heterogeneous products and low yields following production, due in part to complex structures and multiple glycosylation sites (9, 11–13).

Studies over the last several years have revealed that glycosylation, both N-linked and O-linked, can play profound roles in modifying the effector functions for each antibody class. This ranges from maintaining the structure, tuning effector functions, engaging co-receptors and co-factors, improving their stability, and modifying antigenicity (14, 15). In this review, we will focus on the impact of glycosylation across the antibody classes, with a particular focus on the impact of sialic acid.

## Antibody N-Linked and O-Linked Glycosylation

Glycosylation of antibodies is initiated in the endoplasmic reticulum (ER) of antibody-producing cells and further trimming and remodeling steps are processed by glycosidases and glycosyltransferases in the Golgi apparatus (16). N-linked glycosylation occurs on the amino acid asparagine within the consensus sequence of Asn-X-Ser/Thr, where X is any amino acid except proline. The glycosylation process starts with the transfer of the preassembled, lipid-linked glycan precursor, triglucosylated high-mannose-type tetradecasaccharide, to the asparagine residue by oligosaccharyl transferase in the ER (17). The glycan precursor undergoes further processing by glycosidases, which remove the glucose residues, one mannose residue, and three N-acetylglucosamine (GlcNAc) residues. Once the antibody is translocated into the cis-Golgi, the glycan undergoes further trimming by glycosidases and further remodeling is performed by the glycosyltransferases in the median Golgi.

Based on their composition, glycans are classified into three types: oligomannose, hybrid, and complex (**Figure 1B**) (16–18). Oligomannose glycans contain 5 to 9 branching mannose residues which are not further cleaved during processing. Complex glycans possess a core structure composed of two inner GlcNAc residues, three mannose residues, and two outer GlcNAc residues each β-1,2-linked to the α-3 and the α-6 mannose residues, forming two antennae (19). The innermost GlcNAc can be further modified by addition of an α-1,6 linked fucose, which is catalyzed by α-1,6 fucosyltransferase (FUT8) (16, 20). One galactose residue may be added to each of the β-1,2 GlcNAc by β -1,4 galactosyl transferase-1, and this addition can be further extended by the addition of sialic acid by α-2,6 sialyltransferase (16, 21). Hybrid type glycans are a combination of the high-mannose and complex types, with one antenna possessing only mannose residues and the other arm including one GlcNAc residue with further addition of galactose and then sialic acid. Variations in antibody glycosylation depend on substrate availability, steric hindrance, and expression level of glycosyltransferases (22). This leads to heterogeneity in glycosylation, which the immune system uses to fine-tune immune responses, and certain glycosylation patterns can be used as biomarkers for certain diseases.

O-linked glycosylation is present in the hinge region of IgA1, IgG3, and in the Fc portions of human IgD (23–25). O-linked glycosylation occurs on serine and threonine residues, but the consensus sequence for this modification has not yet been identified, the regulation of O-linked glycosylation is less well understood compared to the N-linked pathways, and it occurs exclusively in the Golgi. In the first step, GalNAc is attached to serine or threonine by UDP-N-acetylgalactosaminyl transferase 2 (GalNAc-T2) in the *cis*-Golgi. Next, the addition of galactose is catalyzed by β1,3-galactosyltransferase (C1GalT1), an enzyme which also requires the molecular chaperone COSMC for stability and function. O-linked glycans are further extended by the addition of sialic acid to the GalNAc and/or galactose residues by α2,6-sialyltransferase (ST6GalNAc-I)or α2,3-sialyltransferase (ST3Gal-1), respectively (26–29) in *medial*- and *trans*-Golgi.

## Antibody Structure, Glycosylation Across Classes and Subclasses

### IgG Antibodies

The monomeric structures of all antibodies, including IgG, contain two functional domains. The antigen-binding region (Fab) is involved in the recognition of antigens and the fragment crystallizable region (Fc) interacts with FcγRs and other effector proteins, including complement component C1q to mediate effector functions. The Fab region is composed of one variable and one constant domain (CH1) of both the heavy and light chains, while the Fc domain is a homodimer consisting of the heavy chain constant domains (CH2 and CH3). A flexible hinge region connects the Fab and Fc domains.

The four subclasses of IgG antibodies are numbered based on their prevalence in the serum: IgG1, IgG2, IgG3, and IgG4 (30–32). These four IgG subclasses share more than 90% sequence similarity, but each subclass has a distinct functional profile in mediating effector function, complement activation, half-life, and placental transport (33, 34). The Fc domain of each subclass carries a conserved N-linked glycosylation site at asparagine 297 (N297) in the CH2 domain. The glycan occupying this site has a complex biantennary structure. Several studies have shown roles for Fc glycosylation in mediating immune effector functions, including anti-inflammatory responses, ADCC, CDC, and antibody-dependent cell-mediated phagocytosis (ADCP) (34–38). Structural studies showed Fc glycans play a crucial role in maintaining an open conformation of the Fc domain and mediating interaction with FcγRs and C1q (39, 40). Removal of this glycosylation site abrogates effector functions and abolishes binding to FcγRs and C1q (41–43). Intriguingly, IgG Fc glycans are highly heterogeneous with more than 30 distinct glycoforms detected on IgG in healthy individuals (44, 45). The composition of the IgG Fc glycan has been demonstrated to significantly impact effector functions, pharmacokinetics, immunogenicity, stability, and aggregation (46–48).

#### IgG Sialylation

**Figure 2:**
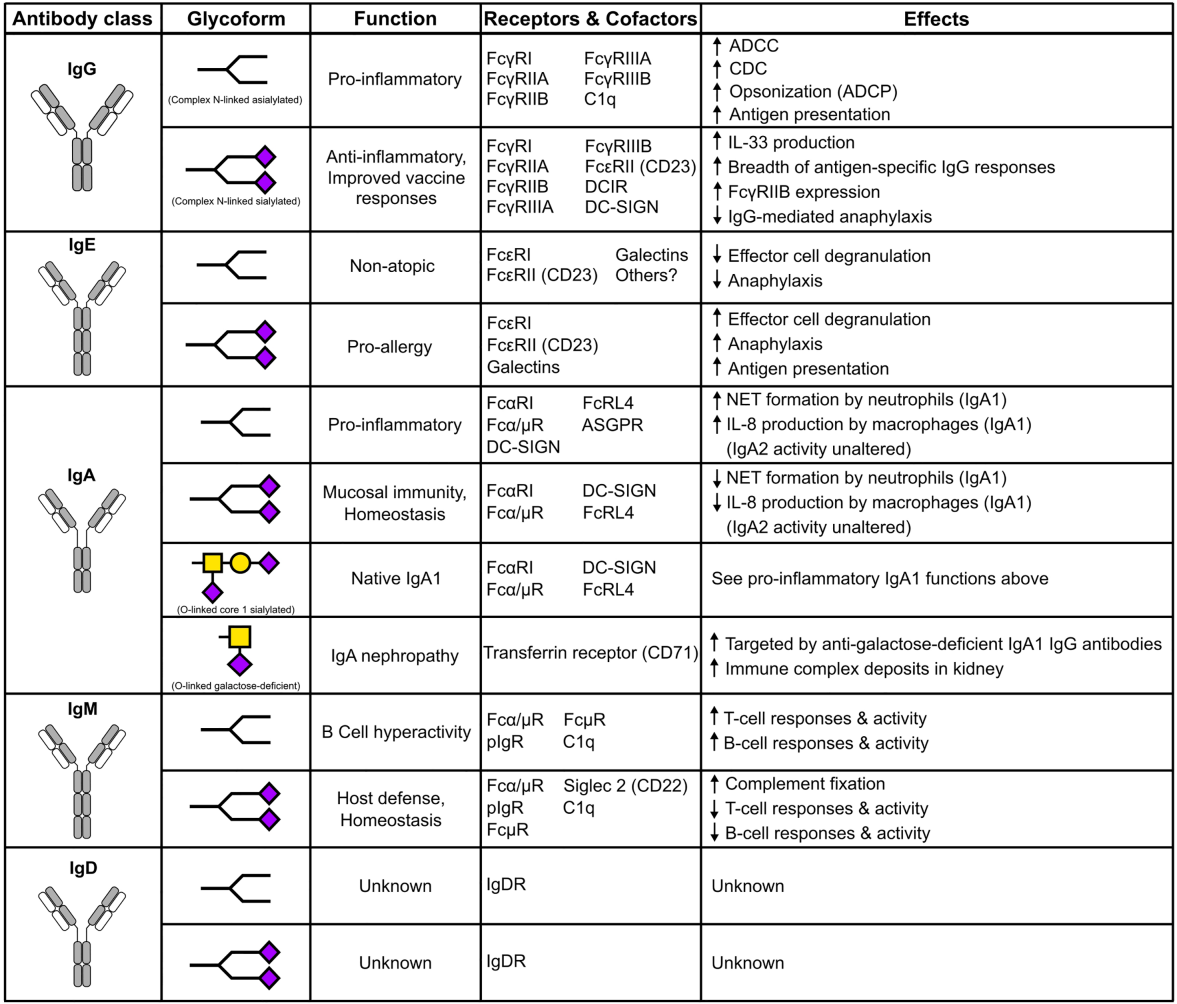
The impact of N- and O-linked sialylation across antibody classes. Antibody schematics are shown, along with skeleton sialylated (terminating in purple diamonds) or asialylated N-linked glycans. O-glycans and IgA1 are comprised of GalNAc (Yellow squares), galactose (yellow circles), and sialic acid (purple diamonds).

In a healthy individuals, approximately 10-15% of serum IgG are sialylated, with the majority of those possessing monosialylated glycoforms (49). Indeed, this percentage is reduced during inflammation and autoimmune disease flares (49, 50). IVIG is a therapeutic preparation of monomeric IgG pooled from the plasma of thousands of donors. It is administered as an IgG-replacement to immunocompromised individuals at 400-600mg/kg. Paradoxically, it is used to treat autoimmune and inflammatory diseases such as Myasthenia gravis (MG), immune thrombocytopenia (ITP), multiple sclerosis (MS), systemic lupus erythematosus (SLE), chronic inflammatory demyelinating neuropathy, and Kawasaki disease at a high dose of 1-2g/kg (51–53). Multiple studies support α-2,6 sialylated IgG as the biologically active component of immunomodulatory high dose IVIG (**Figure 2**) (36, 37, 54, 55). A series of mechanistic studies revealed that the immunomodulatory and anti-inflammatory activity of IVIG requires the inhibitory receptor FcγRIIB, and that FcγRIIB surface expression is increased on macrophages, dendritic cells, and B cells following IVIG infusion (56–58).

Further studies revealed terminal α-2,6 sialic acid on the IgG Fc glycans were required for anti-inflammatory activity, and C type lectins Specific ICAM-3 Grabbing Non-Integrin-Related 1 (SIGN-R1) and Dendritic Cell-Specific ICAM-3 Grabbing Non-Integrin (DC-SIGN) in mice and humans, respectively, were also required (36, 37, 39, 54). Administration of sialylated IgG Fc induces IL-33 production, which in turn triggers the expansion of IL-4-producing basophils and T regulatory cells, culminating in upregulation of FcγRIIB expression on effector cells (59, 60). While functional models support a role for DC-SIGN in the anti-inflammatory activity of sialylated IgG, whether there is a direct interaction between these entities is under debate (61, 62). Insertion of additional N-linked glycosylation sites in the hinge region of IgG1 Fc *via* mutants D221N/N297A/N563A and D221N/N563A increases sialyation 75% and 44% respectively, resulting in increased binding to Siglec-1 (Sialoadhesion), and these mutants also inhibited demyelination by anti-MOG antibodies in an *ex vivo* model when multimerized (63–65). Another study demonstrated that sialylated IVIG mediates anti-inflammatory activity by induction of T regulatory cells through engagement of the dendritic cell immunoreceptor (DCIR) (66).

IgG sialylation is emerging as a therapeutic option to treat autoimmune diseases. A phase 1/2 clinical trial (NCT03866577) reported sialylated IVIG offered protection to patients with ITP (67). Confirmation of previous murine studies came following IVIG infusion in chronic inflammatory demyelinating polyneuropathy (CIDP) patients, which led to increased surface expression of FcγRIIB (68, 69). Further, IL-33 has been detected in the serum following IVIG infusion (70). Finally, sialylated IgG attenuated IgG-mediated anaphylaxis in murine models, but additional experiments are required to determine whether sialylated IgG is a viable therapeutic for allergic disease (71, 72).

While sialylation conveys anti-inflammatory activity to monomeric IgG, it imparts unique functions to antigen-specific IgG. Indeed, increased antigen-specific IgG was detected following vaccination to *Streptococcus pneumonia* and SARS-CoV-2, and administration of sialylated IgG immune complexes led to an enhanced breadth of IgG responses in a FcϵRII (CD23)-dependent manner (73–75). IgG sialylation appears to have little impact on ADCC, or FcγRI and FcγRIIIA binding, and a only small enhancement of binding to FcγRIIA (76). Some functional studies have reported that sialylation impairs IgG-mediated ADCC and CDC (77–80). Consistently, asialylated IgG1 displayed enhanced C1q binding and CDC activity compared to sialylated IgG1 (80). In fact, a randomized clinical trial of patients with CIDP receiving high dose IVIG showed increased IgG sialyation and significantly lower levels of complement activation associated with disease remission (80). One contrasting study reported IgG1 Fc sialylation resulted in increased C1q binding (81). Further characterization of the role of IgG Fc sialylation and other glycoforms in FcγR binding, C1q binding, and functional assays is needed to resolve some of these discrepancies.

#### IgG Fucosylation, Galactosylation, and Bisecting GlcNAc

It is well-established that non-sialic acid sugars also exert important and profound influence over IgG effector functions, as has been reviewed extensively elsewhere (82–85). Briefly, more than 90% of IgG glycans in human serum contain a fucose residue attached to the innermost GlcNAc core structure (86). Antibody fucosylation is catalyzed by FUT8 in the *medial/trans*-Golgi. Several studies have demonstrated that removal of the core fucose residue increases IgG binding affinity to the human FcγRIIIA receptor expressed on macrophages, dendritic cells, and natural killer cells, resulting in enhanced ADCC (87, 88). This generated tremendous interest in the roles of IgG glycosylation in Fc receptor binding and antibody function. Afucosylated therapeutic antibodies, including anti-HER2 and anti-CD20 IgG1, show 100-fold higher ADCC compared to fucosylated anti-HER2 antibodies (89). Thus, there is a huge potential in immunotherapy for afucosylated antibodies with increased ADCC activity (90–92). Further, the severity of viral diseases caused by Dengue virus and SARS-CoV-2 are associated with the percentage and titers of viral-specific afucosylated IgG (93, 94).

The IgG N-glycan may be agalactosylated (G0F), mono-galactosylated (G1F), or di-galactosylated (G2F), and the effector functions of each glycoform can vary significantly. In the serum of a healthy individual, G0F, G1F, and G2F each account for approximately 35%, 35%, and 15% of IgG glycoforms, respectively (49, 95). A decrease in IgG galactosylation in human serum is associated with chronic inflammatory and autoimmune diseases, such as SLE, MS, rheumatoid arthritis (RA), auto-immune vasculitis, active spondyloarthropathy, Crohn’s disease, inflammatory bowel disease, and psoriatic arthritis (49, 96–100). IgG galactosylation level also decreases with age and inflammation, but the rate of change is faster during the development of inflammatory conditions compared to typical aging (49). Galactosylated IgG1 immune complexes showed anti-inflammatory activity in mice through interactions with FcγRIIB and the receptor Dectin-1 (101). Galactosylated IgG have also been shown to bind complement effectively (102).

In healthy individuals, only a small fraction (10-15%) of antibodies contain bisecting GlcNAc (biGlcNAc) (49). In the Golgi, β-1,4-N-acetylglucosaminyltransferase III (GnT-III) catalyzes the addition of biGlcNAc to the internal mannose residue. Recombinant IgG Fcs with biGlcNAc possess an increase in ADCC activity and FcγRIIIA binding (103). However, the addition of biGlcNAc inhibits the addition of a core fucose residue due to steric hinderance, and increased ADCC is also attributed to the inhibition of fucose attachment (83).

### IgE Antibodies

In comparison with other antibodies, IgE is the least abundant antibody in serum with a concentration of 50–100 ng/ml, and a half-life of only two to three days; binding to the high affinity receptor FcϵRI expressed on mast cells extends tissue half-life to three weeks (104). IgE is involved in triggering allergic reactions to food and pollen, and is involved in atopic diseases such as asthma and atopic dermatitis. The incidence of allergic disease is significantly increasing worldwide, with an estimated of 30-40% of the population having one or more allergic diseases. Thus, there has been significant interest in developing IgE-targeted therapeutic antibodies to treat IgE-mediated allergies. For example, Xolair (Omalizumab) is an FDA approved monoclonal antibody to treat IgE-mediated allergic disease. On the other hand, IgE antibodies also provide a defense against parasite infections and insect venoms (105–107).

IgE is composed of two identical heavy chains and two light chains. Each heavy chain consists of a single variable domain and four constant domains, and the light chain is composed of one variable and one constant domain. IgE is a heavily glycosylated monomeric antibody, with seven N-linked glycosylation sites spread across the four constant domains of the heavy chain. N394 is a conserved glycosylation site with an oligomannose glycan, N383 is unoccupied, and the other five sites (N140, N168, N218, N265, N371) are occupied by complex type biantennary glycans (108–111). The major structural differences of IgE compared to IgG are a shorter hinge region, the presence of an extra constant region, and an increased number of glycosylation sites.

Similar to IgG, the constant region of IgE mediates various effector functions through binding to the high-affinity and low-affinity receptors expressed on innate immune cells. The IgE high affinity receptor FcϵRI is expressed on mast cells and basophils shows a significantly high binding affinity, approximately 10^11^ M^−1^ (112). The binding of an allergen to IgE followed by crosslinking the FcϵRI receptors triggers cellular degranulation and symptoms of allergic inflammation (113). The low-affinity IgE receptor, FcεRII (CD23), has a binding affinity coefficient of 10^6^ M^-1^. Interactions with FcεRI or FcεRII depend on IgE’s Fc conformation. In the open conformation, IgE binds to FcϵRI at the CH3 domain, while in the closed conformation IgE binds to FcεRII at both the CH3 and CH4 domains (114, 115). Binding to the high affinity and low affinity receptors is mutually exclusive; conformation changes upon binding to one receptor hinder the binding to another receptor (116).

#### IgE Sialylation

With the emergence of glycosylation’s role in regulating IgG structure and function, there is growing interest in the role of glycosylation across other antibody classes. Initial work indicated that IgE glycosylation is critical for FcϵRI binding and effector functions (117–119). Subsequently, other studies showed that aglycosylated IgE produced in *E. coli* binds to FcϵRI and can trigger effector functions, suggesting a null effect of glycosylation (120–122). However, studies coupling *in vitro*, cell-based, and mice model experiments have shown an important role for IgE glycosylation. The point mutation N384Q in murine IgE (mIgE), equivalent to human N383Q, abolished binding to FcϵRI in ear mast cells and cell-based assays, and similar results were also observed with the enzymatic treatment of mIgE using PNGaseF to remove all N-glycans (123). These results suggest an absolute requirement of mIgE N384 glycosylation for the initiation of anaphylaxis. Further, circular dichroism results showed enzymatic removal of N-linked glycans altered the secondary structure of IgE and abrogated binding to FcϵRI (123). These results highlight the role of glycosylation in binding to the high-affinity receptor similar to the role of IgG glycosylation in FcγR binding. This also suggests that IgE produced in *E. coli* might adopt a different conformation when refolding than a glycosylated IgE and thus can still result in FcϵRI binding.

Disease-specific IgE glycosylation patterns have been characterized in allergic and atopic cohorts (111). Between the two cohorts, mannose, fucose, and biGlcNAc content in complex bi-antennary type glycans were largely similar. However, terminal galactose was enriched on atopic total IgE while increased sialylation was observed in allergic IgE. Functional experiments revealed increased mast cell degranulation was observed after sensitization with peanut-allergic IgE compared to the atopic IgE. The role of IgE sialylation was further confirmed by the passive cutaneous anaphylaxis model. Indeed, sialylated IgE increased anaphylaxis compared to asialylated IgE (111). The emerging patterns of the effects of antibody sialylation are intriguing; IgG sialylation mediates anti-inflammatory activity, but IgE sialyation promotes allergy (**Figure 2**).

### IgM Antibodies

There are generally thought to be two types of IgM produced, called natural IgM and immune IgM. Natural IgM antibodies are produced spontaneously and act as the first line of defense during microbial infections, prior to the adaptive immune response; its repertoire is largely unaffected by external antigens (124, 125). In humans, natural IgM antibodies are produced by B1 cells without exposure to exogenous antigens, are polyreactive, and constitute the majority of circulating IgM in serum (126, 127). Natural IgM antibodies can recognize specific neoepitopes on apoptotic cells to remove them selectively and to maintain tissue homeostasis. Antigens recognized by natural IgM include specific carbohydrates, phospholipids, and double-stranded DNA (128–130). The other IgM class, immune IgM, is produced after exposure to external antigens (131). While the source of the two IgM classes is different, the differences in their structural and functional properties are minimal (132).

IgM antibodies are found in a pentamer or hexamer format, in which each monomer is composed of a heavy chain with one variable region and four constant regions (CH1, CH2, CH3, CH4), and a light chain (133). In contrast to IgG, the molecular structure of IgM possesses a short hinge region, similar to IgE. A short peptide sequence of 18 amino acids, called a tailpiece, is essential for IgM multimerization. The tailpiece of each heavy chain forms disulfide bonds with each heavy chain monomer to form IgM and another disulfide bond between C414 residues in the CH3 region of each heavy chain is involved in the formation of the multimer (134, 135). Tailpiece multimerization has also been successfully used to form IgG hexamers (136, 137). IgM also contains a J chain in the pentameric structure which is involved in the transportation of IgM through binding to specific receptors (9, 138, 139).

Apart from multimerization, glycosylation sites add further complexity to the IgM structure. IgM is heavily glycosylated in comparison with IgG; it has five N-linked glycosylation sites in the heavy chain (N171 (CH1), N332 (CH2), N395, N402 (both in CH3)), one in the tailpiece (N563), and one on the J chain (140, 141). The N-linked glycosylation pattern of IgM includes complex type and high-mannose type glycans; as shown in **Figure 1A**, N171, N332, and N395 are occupied by complex type glycans and N402 and N563 contain high-mannose type glycans (141, 142). Similar to IgG, IgM complex type glycans also may contain terminal sialic acid.

#### IgM Sialylation

Natural anti-T lymphocyte IgM antibody levels are increased in patients with inflammatory conditions, HIV infections, and SLE compared to healthy individuals (143, 144). These antibodies mediate the inhibitory effects on anti-CD3–induced T cell activation but for a time the mechanism was not completely understood (145, 146). A recent study by *Colucci et al.* shows that IgM’s inhibitory effects depend on antibody glycosylation (147). Five different IgM antibodies purified from healthy donors (n=2) or myeloma patients (n=3) were incubated with human PBMCs. Results showed that the IgM antibody from healthy donors (IgMh) bound to the T cells and were then internalized and inhibited T cell proliferation. Meanwhile, IgM from myeloma patients (IgMm) remained on the surface and did not inhibit T cell proliferation. Glycosylation analysis of these antibodies showed only the IgMh, which mediated the inhibitory effects, contained sialic acid but the IgMm antibodies did not. Furthermore, removal of sialic acid using neuraminidase enzyme completely abrogated internalization of IgM and this asialylated IgM did not inhibit T-cell proliferation. The proposed mechanism is that both sialylated and asialylated IgM bind to FcμR, but only the sialylated antibody is internalized and triggers the inhibitory pathways (147, 148).

### IgA Antibodies

In the serum, IgA is the second most prevalent circulating antibody after IgG and it is the predominant antibody found in external secretions such as those that bathe mucosal surfaces (8). Interestingly, the molecular form of IgA varies between the serum and mucosal surfaces. In the serum IgA exists in the monomeric form, but on mucosal surfaces IgA exists as a dimer called secretory IgA (SIgA). SIgA contains two IgA molecules joined by a J chain, similarly to IgM, to form a dimer, and this complex is also bound to a secretory component (149). Serum IgA contains two heavy chains composed of one variable region and three constant regions, and two light chains.

Based on its structure, IgA is divided into subclasses IgA1 and IgA2. IgA1 contains a hinge region with six O-linked glycosylation sites and two N-Linked glycosylation sites on each heavy chain as shown in **Figure 1A**. In contrast, IgA2 lacks O-linked glycosylation due to a shortened hinge region and has two N-linked glycosylation sites per heavy chain. The glycosylation pattern of the O-linked glycans may vary, but the majority of the O-linked glycans contain GalNAc and galactose with one or two sialic acids (11, 26).

IgA blocks the entry of pathogens through antigen-binding sites and the IgA-specific receptor FcαRI mediates the clearance mechanism (150). The SIgA molecule, with its multiple antigen-binding sites, provides high avidity to cross-link antigens in a highly efficient manner, which can block pathogen activity. Both serum IgA and secretory SIgA bind to the FcαRI receptor with similar binding affinity but cause divergent downstream effects. To evade the immune system, pathogenic bacteria such as *Staphylococcus aureus* produce IgA binding proteins to inhibit IgA and FcαRI (151). In another strategy, pathogenic bacteria produce specific bacterial proteases against IgA, which specifically cleaves inside the hinge region of IgA leading to the inefficient clearance of pathogens (11, 150).

Several studies have shown that variations in IgA1 O-linked glycosylation play a crucial role in the pathogenesis of IgA Nephropathy (IgAN) and kidney failure (152). Analysis of IgAN patient samples showed a galactose deficiency in O-linked glycans, resulting in immune complex formation stimulated by anti-IgA1 IgG antibodies specific for this O-linked glycan. The large immune complexes escape clearance mechanisms and deposit in the renal mesangium, leading to glomerular injury (26, 152–154). As mentioned previously, glycosylation patterns are driven by the availability of substrate and enzyme expression during biosynthesis. In the case of a IgAN, there is deficiency in the galactosyltransferase enzyme C1GALT1, resulting in IgA1 with galactose-deficient O-linked glycosylation in the hinge region (26).

#### IgA Sialylation

Recent studies have shown IgA subclasses isolated from serum display different effector functions (**Figure 2**). IgA2 elicits pro-inflammatory effects on macrophages and neutrophils, but this is not observed with IgA1. These differences are attributed to variations in N-linked glycosylation, where IgA2 has 25% less sialylation compared to IgA1. These results were further confirmed by the enzymatic removal of total sialic acid using neuraminidase and the complete removal of N-linked glycans using PNGaseF, both of which resulted in increased pro-inflammatory activity (155). Another study showed IgA mediates anti-viral activity through sialic acid from the complex N-linked glycan at N459, where sialic acid interfered with cell surface attachment of influenza A (156).

## Glycoengineering

Monoclonal antibody (mAb) based therapeutics are the fastest-growing group of therapeutics for several clinical indications including oncology, autoimmune diseases, inflammatory disease, and bacterial and viral infections. This field is dominated by IgG-based therapeutics. However, IgE has been developed as an immunotherpeautic agent for cancer, and IgA-based therapeutics have been proposed for treatment of bacterial and viral infections, and as anti-inflammatory and anti-tumor agents (10, 11, 150, 157). More testing is needed to determine the best suited glycoforms for these antibody classes. Much of the glycoengineering focus thus far has revolved around generation of afucosylated IgG, which is reviewed extensively elsewhere (82, 92, 158, 159).

Mammalian host expression systems are used for the production of antibodies to maintain mammalian antibody glycosylation patterns and minimize immunogenicity. However, there are differences in glycosylation patterns between endogenous antibodies produced in humans and mammalian cell lines. Antibody glycosylation is influenced by the host cell, glycosyltransferase expression, and cell culture conditions, such as low dissolved oxygen concentration and a reduced culture reduction potential (160, 161). Glycoforms of therapeutic mAbs produced in CHO, HEK293, mouse myeloma NS0, and Sp2/0 cell lines are heterogeneous with a predominance of the G0F glycoform produced, along with limited galactosylated and sialylated glycoforms. Therapeutic mAbs produced in CHO cells are less galactosylated compared to mAbs produced in mouse myeloma cell lines (162). The rat hybridoma cell line YB2/0 has low α-1,6 fucosyltransferase activity; therefore, this cell line is used for the production of mAbs with lower fucosylation levels. Specific glycosylation patterns, such as the absence of fucose, bisecting GlcNAc, and the presence of high mannose glycans increase ADCC, while galactose increases CDCC and sialic acid increases anti-inflammatory activity (163–167). Since different antibody glycoforms have distinct effects on biological activity, the production of homogenous glycoforms is necessary to improve therapeutic outcomes and maintain treatment quality. Enrichment of specific glycoforms and separation of said glycoforms using chromatography is challenging. Alternatively, two strategies have been employed to generate homogenous glycoforms with tailor-made sugar residues to modify antibody function. Host cell glycosylation pathway manipulation, called mammalian cell line glycoengineering, and *in vitro* glycoengineering are widely used to produce homogeneously glycosylated antibodies (91, 92, 158).

### Mammalian Cell Line Engineering

**Figure 3:**
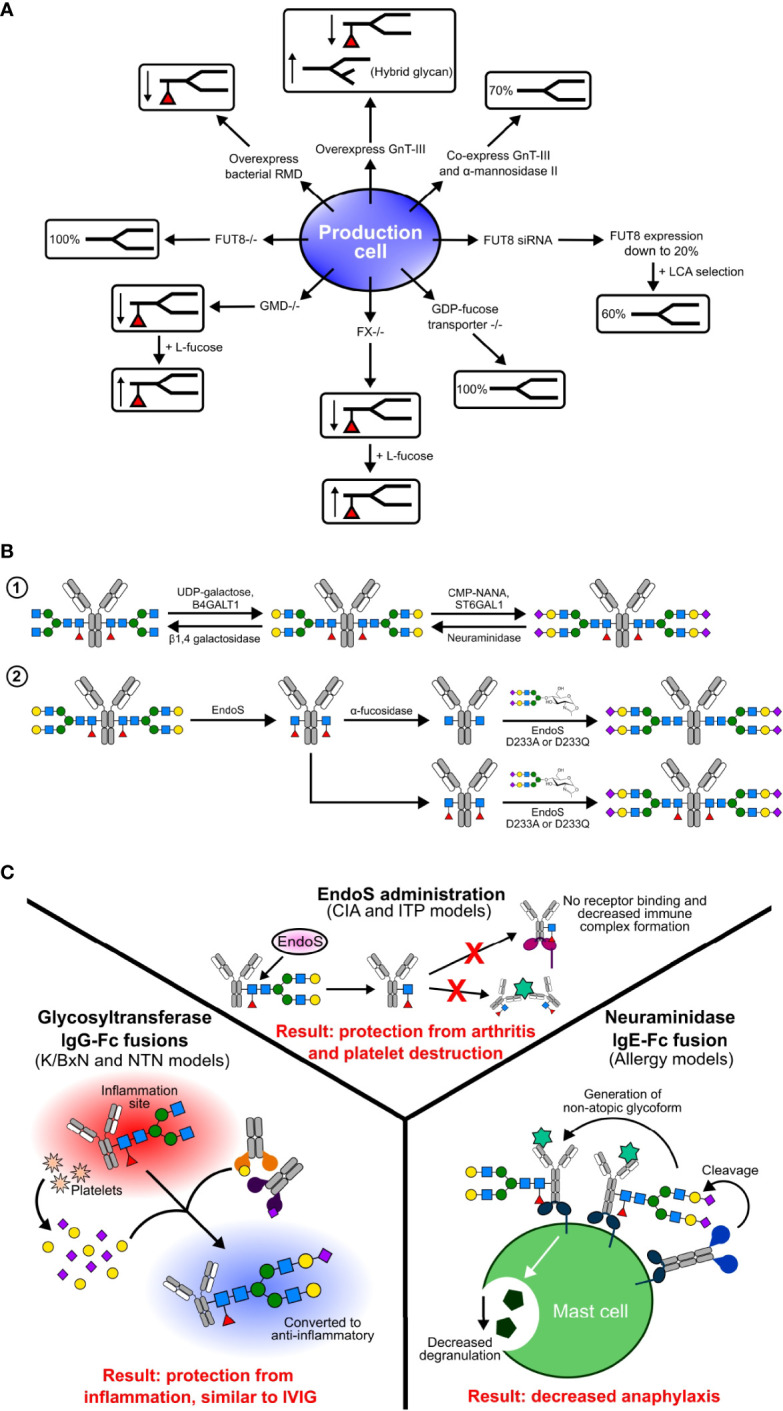
Glycoengineering methods in production cell lines **(A)**, *in vitro* **(B)**, and *in vivo* **(C)**. **(A)** Various genetic manipulations have been performed in production cells to produce differentially fucosylated antibodies. In some cell lines, such as GMD and FX knockouts, fucosylation can be rescued by the addition of substrates such as L-fucose. **(B)** *In vitro* methods of glycoengineering and sialylation include step-wise enzymatic building of glycans and chemoenzymatic engineering. **(C)** *In vivo* glycoengineering involves administration of enzymes, and have had success in some pre-clinical animal models and on human cells.

Biosynthetic pathways in mammalian cell lines can be manipulated to produce afucosylated antibodies (**Figure 3A**). This strategy has focused on non-sialylated glycoforms by overexpression of different enzymes, generating gene knockout lines, and using monosaccharides as inhibitors (82, 92, 158). Recombinant glycoproteins and antibodies produced in CHO cells only produce α-2,3 sialylated glycans due to lack of ST6GAL1 gene expression. To increase sialylation, different strategies have been developed, including overexpression of the CMP-sialic acid transporter, overexpression of sialyltransferases, and knock out of genes (168–171). Production cell lines engineered to overexpress trans-Golgi enzymes B4GALT1 and ST6GAL1 have been described to generate IgG with enriched ~70% biantennary sialylated glycoforms (169). Co-expression of B4GALT1 and ST6GAL1 with IgG increased the amount of sialylation to 32% (170). Using gene editing technology, a CHO^-Mgat2/-Stgal4/-Stgal6/+B4GALT1/+ST6GAL1^ clone was generated by knocking out the ST3GAL4, ST3GAL6, and Mgat2 genes to inhibit α-2,3 sialylation and knocked in the B4GALT1 and ST6GAL1 genes to increase α-2,6 sialylation (171).

Antibody fucosylation is decreased by overexpression of GnT-III. GnT-III is expressed in the Golgi and catalyzes the addition of biGlcNAc on to a β-linked mannose residue of an N-linked glycan. The addition of biGlcNAc increases steric hindrance, inhibits the addition of fucose, and produces hybrid type N-glycans. Although CHO cells do not express GnT-III, overexpression of it in CHO cells resulted in reduced antibody Fc fucosylation due to the addition of biGlcNAc (103, 172). This method is further improved by co-expression of GnT-III and Golgi α-mannosidase II, which results in a 70% decrease in core fucosylation (103, 173).

Gene knock out cell lines were developed by targeting specific genes involved in core fucosylation. The FUT8 gene encoding α-1,6 fucosyltransferase catalyzes the addition of α-1,6 fucose to the innermost GlcNAc. In the CHO-DG44 cell line, small interfering RNAs (siRNAs) targeting FUT8 reduced mRNA expression levels to 20%. Coupling this method with *Lens culinaris agglutinin* (LCA) selection produced 60% afucosylated antibodies with a resulting 100-fold increase in ADCC (174).

Afucosylated antibodies can also be generated by manipulation of GDP-fucose biosynthetic pathways (175). GDP-mannose 4,6-dehydratase (GMD) and GDP-4-keto-6-deoxymannose-3,5-epimerase-4-reductase (FX) catalyze the conversion of GDP-mannose into GDP-fucose. In knockout lines for each of these enzymes, core fucosylation is absent due to a lack of GDP-fucose but can be rescued by addition of L-fucose to the media (176, 177). Alternatively, overexpression of the bacterial GDP-6-deoxy-D-lyxo-4-hexulose reductase (RMD) enzyme reduces core fucosylation by depleting the intermediate GDP-4-keto-6-deoxy-D-mannose used in the synthesis GDP-fucose (175, 178, 179). An additional strategy to generate afucosylated IgG involves the addition of fucose analogs 2-fluorofucose and 5-alkynylfucose to inhibit the biosynthetic pathway of fucosylation (180).

### *In Vitro* Glycoengineering

There is a tremendous improvement in the generation of glycoengineered antibodies through biosynthetic pathways and genetic manipulations in mammalian and non-mammalian cells. However, these methods require complex protocols and special cell line development for each type of glycan addition or deletion. These problems can be overcome by using *in vitro* glycoengineering, where trimming or extending of antibody glycans is performed using substrates, glycosyltransferases, and glycosidases (**Figure 3B**). These approaches have been successfully applied to antibodies generated in both mammalian and non-mammalian cells. There are two types of *in vitro* glycoengineering methods: enzymatic and chemoenzymatic.

In the enzymatic method, the antibody of interest is treated with uridine diphosphate galactose (UDP-Gal) and β-1,4 galactosyltransferase-1 (B4GalT1) to generate the G2F glycoform, which is further modified by treating the product with cytidine 5′ mono-phospho-N-acetyl neuraminic acid (CMP-NANA) and α 2,6 sialyltransferase (ST6Gal1) to produce the G2S2F glycoform (55). This method can also be applied to remove sialic acid and galactose by treatment with neuraminidase and β-1,4 galactosidase. In early studies, lectin enrichment was used to prepare sialylated Fc, but later studies used enzymatic glycoengineering methods to add galactose followed by sialic acid (36, 37). Several studies have shown sialylated IgG1 Fc and sialylated IVIG protect mice from induced arthritis at lower doses compared to IVIG (37, 55). This data is further supported by the use of sialylated IgG Fc clinical trials for the successful treatment of ITP (67). This method is limited by the stepwise addition of sugars.

Chemoenzymatic glycoengineering of IgG antibodies is a two-step process, consisting first of deglycosylation of N-linked glycans using native endoglycosidases followed by the addition of homogenous N-linked glycans using mutant endoglycosidases. This strategy was improved by the discovery of the novel endoglycosidase endo-β-N-acetylglucosaminidase (EndoS) mutants D233A and EndoS D233Q, which allowed the addition of N-linked glycans to GlcNAc or core-fucosylated GlcNAc (181). This is a very robust method that allows for the addition of the entire glycan complex at one time. Using the chemoenzymatic method, Rituximab has been glycoengineered to prepare homogenous G2, S2G2, G2F, and S2G2F glycoforms. Results from *in vivo* and *in vitro* studies have shown that only afucosylated glycoforms increased FcγRIIIA binding and elevated ADCC compared to the G2F and G2S2F glycoforms (77). Enzymatic glycoengineering and biosynthetic pathway manipulation are limited to the addition of complex biantennary type glycans, but the chemoenzymatic method allows selective addition of branched glycans. The D165A and D165Q mutants of the bacterial endoglycosidase Endo-F3 allow selective addition of bi- and triantennary N-glycans to the fucose core (182). The Endo-F3 D165A and Endo-S D233A allow site-specific glycoengineering of the Fab and Fc domains, respectively (183).

Currently, antibody glycoengineering efforts are exclusively focused on the Fc-core due to its role in mediating effector functions (63, 77, 82), but it should be noted that the antibody Fab domain also undergoes glycosylation for antigen recognition and other functions (184–186). The therapeutic antibody Cetuximab undergoes glycosylation in the Fab domain at N88 and in the Fc at N297. Glycoengineered Cetuximab without fucose at the Fc core and with sialic acid at Fab region showed increased binding affinity to FcγRIIIA and increased ADCC (183). Other applications of chemoenzymatic methods include bioconjugation, site specific labelling, and the synthesis of antibody-drug conjugates.

### *In Vivo* Glycoengineering

Biosynthetic pathway manipulation of cell lines requires more time to establish knock out cell lines but is faster once the cell line is established, whereas *in vitro* glycoengineering methods require more time and effort in the purification steps. Another emerging strategy is glycoengineering circulating IgG antibodies *in vivo* using glycosidases and glycosyltransferases (**Figure 3C**). The first time deglycosylation of circulating IgG antibodies was shown was by *in vivo* administration of EndoS from the human pathogen *Streptococcus pyogenes (* 187). EndoS selectively catalyzes the hydrolysis of the glycosidic bond between the first two N-acetylglucosamine residues of the of the N-linked glycan on the Fc of each IgG subclass (IgG1-4). In the collagen-induced arthritis mouse model, pretreatment of antibodies with EndoS enzyme inhibited the advancement of arthritis, abolished IgG binding to Fc receptors, and disturbed immune complex formation (188). Furthermore, the administration of EndoS has shown a protective effect in a mouse model of IgG-driven ITP (189). However, repeated administration of EndoS triggers an immune response, and using bacterial enzymes in a therapeutic setting raises a safety concern. Alternatively, the bacterial protease IdeS was successful in a trial targeting IgG mediated autoimmune conditions (190–192)

In another strategy of *in vivo* sialylation, human glycosyltransferases B4GalT1 and ST6Gal1 have been fused to IgG Fc to glycoengineer pathogenic antibodies (193). This approach recapitulates sialylated IVIG or IgG anti-inflammatory activity by converting pathogenic antibodies into sialylated, non-pathogenic antibodies. Interestingly, Fc-fused enzymes specifically sialylated pathogenic antibodies only at sites of inflammation, but not the antibodies or other glycoproteins in circulation (193). This specificity of *in vivo* glycoengineering is due to platelets releasing sugar donors, sialic acid and galactose, at sites of inflammation. This strategy has been successfully demonstrated in murine autoimmune disease models, in which Fc-fused enzymes attenuated auto-antibody inflammation by converting autoantibodies into anti-inflammatory mediators (193). By fusing glycoenzymes to IgG Fc, the enzymes’ stability and half-life is increased. *In vivo* sialyation has the potential to be applied to those diseases currently treated using IVIG and offers a potential therapeutic strategy for autoimmune and inflammatory conditions.

Removing sialic acid from IgE is an appealing therapeutic strategy for allergic disease. With that in mind, a bacterial neuraminidase enzyme was fused to the IgE Fc Cϵ2–4 domains (NEUFcϵ), thereby targeting neuraminidase to IgE-bearing cells (111). Administration of this enzyme fusion during murine models of allergic inflammation resulted in attenuation of anaphylaxis (111). However, further testing of modulating antibody sialic acid content *in vivo* is needed.

## Conclusions

Endogenous antibody glycosylation is heterogeneous and varies with age and pathophysiological conditions. Harnessing antibody glycosylation is in its infancy, but applications that are engaged in this endeavor include the development of biologic therapeutics and using certain glycosylation patterns as biomarkers. Therapeutic antibodies are a rapidly growing class for the treatment of cancer, autoimmune diseases, infectious diseases, and other conditions. Glycosylation of therapeutic antibodies is very sensitive to cell culture and other production conditions and variations in glycosylation impacts the quality of the product. Therapeutic antibody glycosylation is a critical quality attribute because of its impact on effector functions and half-life. In depth understanding of IgG glycosylation has revealed the role of each glycan in modulating IgG function and resulted in the development of therapeutic afucosylated and sialylated antibodies.

Glycosylation across antibody classes regulates the structure and function of antibodies. Several studies have shown that sialic acid has divergent functions in antibody classes. For instance, sialylated IgG1 mediates anti-inflammatory activity, sialylated IgE is associated with allergic pathogenicity, sialylated IgA shows anti-viral activity, and sialylated IgM shows inhibitory effects on T-cell proliferation. For most antibody classes, sialylation exerts profound influence over the effector functions. There are multiple, non-mutually exclusive reasons for this, including influencing receptor binding and introducing addition receptors and co-factors. Further studies are required to characterize specific mechanisms of antibody activity and the involvement of additional receptors. Also, the role of sialylation across all antibody subclasses is in need of more examination. *In vitro* sialylation of IgG markedly enriches sialylated glycoform for desire effector functions. This method has been successfully applied to generate sialylated antibodies to mediate anti-inflammatory activity. Indeed, glycoengineering has applicability across antibody classes is a major advantage. More understanding of the role and regulation of antibody sialylation and glycosylation will likely uncover additional applications for glycoengineering.

## Author Contributions

RV, SS, and RA wrote the article. All authors contributed to the article and approved the submitted version.

## Funding

This work was supported by NIH grants (R01 AI155662, R01 AI153441, and R01 AI139669) to RA.

## Conflict of Interest

The authors declare that the research was conducted in the absence of any commercial or financial relationships that could be construed as a potential conflict of interest.

## Publisher’s Note

All claims expressed in this article are solely those of the authors and do not necessarily represent those of their affiliated organizations, or those of the publisher, the editors and the reviewers. Any product that may be evaluated in this article, or claim that may be made by its manufacturer, is not guaranteed or endorsed by the publisher.
